# Oral Biology, Oral Pathology, and Oral Treatments

**DOI:** 10.1155/2016/2849795

**Published:** 2016-08-21

**Authors:** Samir Nammour, Toni Zeinoun, Kenji Yoshida, Aldo Brugnera Junior

**Affiliations:** ^1^Department of Dental Science, Faculty of Medicine, University of Liège, Quai Godfroid Kurth 45, 4020 Liege, Belgium; ^2^Department of Oral and Maxillofacial Surgery, Faculty of Dentistry, Lebanese University, Beirut, Lebanon; ^3^Department of Oral and Maxillofacial Surgery, School of Dentistry, Aichi-Gakuin University, 2-11 Suemori-dori, Chikusa-ku, Nagoya, Aichi-ken 464-8651, Japan; ^4^CITS-Center of Health Technological Innovation and Biomedical Engineering Institute, Unicastelo, São José dos Campos, SP, Brazil

Oral biology, oral pathology, and oral treatments are interesting fields in dentistry. The rapid evolution of technologies and the continuous apparition of new materials and products available for practitioners oblige searchers to evaluate their impact on oral tissues and teeth. The evaluation of the biocompatibility of new products is essential to avoid any tissues damage caused by an eventual toxicity or side effects of therapeutic products or materials.

This special issue is a compendium of different studies and fundamental and clinical researches. Some papers are focused on the microbiological evaluation of the effect of low level laser therapy (LLLT) in peri-implantitis treatment, a new diagnostic approach using microRNAs as salivary markers for periodontal diseases, evaluation of safety irradiation parameters of Nd:YAP laser beam during an in vitro endodontic treatments, a literature overview about the effects of Nd:YAG 1064 nm and diode 810 nm and 980 nm in infected root canals, efficacy of ultrasonic and Er:YAG laser in removing bacteria from the root canal system, a comparative study of microleakage on dental surfaces bonded with three self-etch adhesive systems treated with the Er:YAG laser and bur, and the study of laser Doppler measurement of flow variability in the microcirculation of the palatal mucosa.

We hope that the content of this special issue allows readers to understand the interaction of materials with oral tissues and provides to practitioners new therapeutic methods for their daily practices.

*Samir Nammour* *Toni Zeinoun* *Kenji Yoshida* *Aldo Brugnera Junior*

